# Tryptophan metabolism: a potential link between the gut microbiota and pulmonary vascular remodeling in pulmonary hypertension

**DOI:** 10.3389/fcimb.2026.1960734

**Published:** 2026-09-23

**Authors:** Hui Zhang, Guangyang Ou, Jian Yi, Zhongyi Huang, Lijun Gong, Lan Song, Feiying Wang, Aiguo Dai

**Affiliations:** 1 Clinical Medical College, Hunan University of Chinese Medicine, Changsha, Hunan, China; 2 Respiratory Disease Laboratory, School of Medicine, Hunan University of Chinese Medicine; Key Laboratory of Vascular Biology and Translational Medicine of Hunan Provincial Universities, Changsha, Hunan, China; 3 Medical Innovation Experimental Center, The First Affiliated Hospital of Hunan University of Chinese Medicine, Changsha, Hunan, China; 4 Department of Respiratory Medicine, The First Affiliated Hospital of Hunan University of Chinese Medicine, Changsha, Hunan, China

**Keywords:** gut microbiota, gut–lung axis, indole metabolites, kynurenine, pulmonary hypertension, pulmonary vascular remodeling, serotonin, tryptophan metabolism

## Abstract

Pulmonary hypertension (PH) comprises a group of cardiopulmonary disorders characterized by a sustained increase in pulmonary vascular resistance and pulmonary vascular remodeling, with disease progression potentially leading to right heart failure and death. Current therapies can improve symptoms and hemodynamics in some patients, but effective means of reversing established structural pulmonary vascular remodeling remain lacking. Gut microbial dysbiosis, intestinal barrier injury, and altered microbial metabolism have been proposed to contribute to the development and progression of PH. Tryptophan metabolism is jointly regulated by host tissues, immune cells, and gut microorganisms and serves as a key hub linking the host and its commensal microbiota. The kynurenine, serotonin, and indole pathways may participate in pulmonary vascular remodeling by influencing pulmonary vascular tone, endothelial homeostasis, pulmonary arterial smooth muscle cell phenotype, inflammation, oxidative stress, and cellular metabolism, although the precise mechanisms remain to be clarified. This review systematically summarizes current evidence on the involvement of the gut microbiota and tryptophan metabolism in pulmonary vascular remodeling in PH, with emphasis on the available evidence, potential therapeutic targets, and prospects for clinical translation, thereby providing a basis for further investigation of the metabolic regulation of PH and the development of new intervention strategies.

## 1 Introduction

Pulmonary hypertension (PH) refers to a group of cardiopulmonary disorders with diverse etiologies and pathogenic mechanisms, whose main hemodynamic feature is elevated pulmonary arterial pressure and whose progression may lead to right heart failure or death (Humbert et al., 2022; Mocumbi et al., 2024). PH represents a substantial global health burden and is estimated to affect approximately 1% of the global population, with prevalence increasing markedly with age (Humbert et al., 2022). Contemporary registries report an incidence of PAH of approximately 6 cases per million adults per year and a prevalence of 48–55 cases per million adults (Leber et al., 2021; Humbert et al., 2022). Despite major advances in PAH-targeted therapy, long-term prognosis remains unsatisfactory, with 5-year survival rates ranging from approximately 50% to 86% among adults with PAH (Reinders et al., 2024). According to etiology and hemodynamic characteristics, PH is divided into five groups: group 1, pulmonary arterial hypertension (PAH); group 2, PH associated with left heart disease; group 3, PH associated with lung disease and/or hypoxia; group 4, chronic thromboembolic PH and other pulmonary artery obstructions; and group 5, PH with unclear and/or multifactorial mechanisms (Humbert et al., 2022; Mocumbi et al., 2024). Although these groups differ in etiology, clinical presentation, and the vascular territories involved, pulmonary vascular remodeling is a common pathological basis of disease progression. Interactions among pulmonary arterial endothelial cell dysfunction, abnormal proliferation and migration of pulmonary arterial smooth muscle cells, perivascular inflammation, fibroblast activation, and extracellular matrix deposition ultimately lead to vascular wall thickening, luminal narrowing, and reduced vascular compliance, causing a sustained increase in pulmonary vascular resistance (Morrell et al., 2009; Li et al., 2024). Current management of PH includes treatment of the underlying disease, supportive therapy, targeted pharmacotherapy, surgical or interventional procedures, and lung or heart–lung transplantation for patients with advanced disease (Humbert et al., 2022; Mocumbi et al., 2024). Although these measures can improve symptoms, exercise capacity, and hemodynamics in some patients, effective means of reversing established structural pulmonary vascular remodeling remain unavailable (Humbert et al., 2022; Mocumbi et al., 2024). Further elucidation of the mechanisms that regulate PH and pulmonary vascular remodeling, and the identification of new therapeutic targets, are therefore important.

The concept of the gut–lung axis offers a new perspective on the systemic pathogenesis of PH. The respiratory and gastrointestinal epithelia share an endodermal origin and have similar mucosal barrier and immune defense features (Tichelaar et al., 1999). The gut and lung communicate bidirectionally through circulating, immune, and metabolic signals, allowing changes in the intestinal microenvironment to influence distal lung tissue and pulmonary vascular homeostasis (Yang et al., 2025; Qiu et al., 2026). Clinical studies show that patients with PAH may have changes in gut microbial composition and metabolic function, together with abnormalities in circulating microbiota-related metabolites; some of these features are associated with pulmonary hemodynamic indices and disease severity (Kim et al., 2020; Moutsoglou et al., 2023). Changes in the gut microbiota and metabolic profiles have likewise been observed in different animal models of PH, together with abnormalities in intestinal tissue or barrier function (Chen et al., 2022; Luo et al., 2023). Conversely, PH-associated hypoxia, reduced cardiac output, intestinal hypoperfusion, venous congestion, and systemic inflammation may damage the intestinal epithelial barrier and alter the microbial habitat. Gut abnormalities and pulmonary vascular pathology may therefore form a bidirectional feedback loop, although the mechanisms by which gut-derived signals act on the pulmonary vasculature remain incompletely understood.

Among the metabolic signals that may mediate communication across the gut–lung axis, tryptophan metabolism is jointly regulated by host tissues, immune cells, and gut microorganisms and is therefore of particular interest as a metabolic hub. Tryptophan is metabolized mainly through three pathways: kynurenine, serotonin (5-hydroxytryptamine, 5-HT), and indole (Agus et al., 2018; Roager and Licht, 2018). Bioactive metabolites generated by these pathways can affect pulmonary vascular tone, endothelial homeostasis, pulmonary arterial smooth muscle cell proliferation and migration, inflammation, oxidative stress, and cellular metabolism. Current evidence indicates that altered kynurenine metabolism is associated with PAH severity and clinical outcomes (Jasiewicz et al., 2016; Nagy et al., 2017; Cai et al., 2022; Simpson et al., 2023). 5-HT can promote pulmonary vasoconstriction and smooth muscle cell proliferation through its receptors and transport system, whereas deletion or inhibition of tryptophan hydroxylase 1 attenuates pulmonary vascular remodeling in animal models of PH induced by chronic hypoxia, monocrotaline (MCT), or SU5416/hypoxia (SuHx) (Izikki et al., 2007; Aiello et al., 2017; MacLean, 2018). Gut microorganisms can also convert tryptophan into a range of indole derivatives that influence distant organ function by regulating the intestinal barrier, immune responses, and host receptor signaling (Agus et al., 2018; Roager and Licht, 2018). Together, these findings suggest that tryptophan metabolism may be an important link between changes in the gut microbiota and pulmonary vascular disease.

Although available studies suggest that tryptophan metabolism may be involved in PH-associated pulmonary vascular remodeling, the evidence base differs among pathways, and the origins of the relevant metabolites, their pulmonary vascular target cells, and their causal links to the gut microbiota remain unclear. Accordingly, this review systematically examines the kynurenine, serotonin, and microbiota-related indole pathways and the roles of their metabolites in pulmonary vascular disease, evaluates current evidence for tryptophan metabolism as a potential link between the gut microbiota and pulmonary vascular remodeling, and summarizes related intervention strategies and therapeutic prospects, with the aim of providing a theoretical basis for understanding the metabolic regulation of PH and identifying new targets for pulmonary vascular remodeling. The overall relationships among the gut microbiota, tryptophan metabolism, and pulmonary vascular remodeling are shown in **Figure 1**.

**Figure 1:**
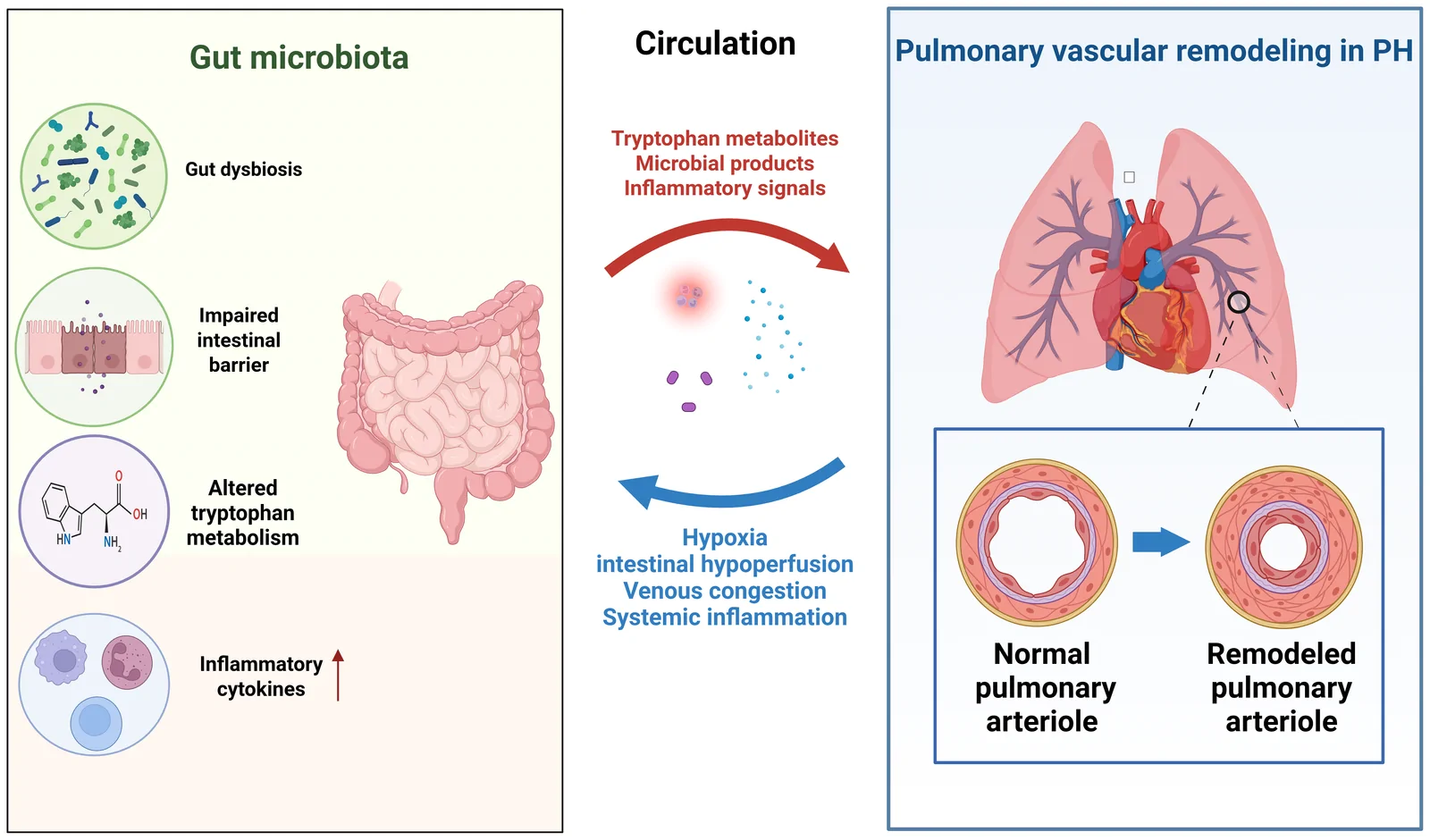
Conceptual framework linking the gut microbiota, tryptophan metabolism, and pulmonary vascular remodeling in pulmonary hypertension. Gut microbial dysbiosis, impaired intestinal barrier integrity, altered tryptophan metabolism, and increased inflammatory signaling may be accompanied by changes in circulating tryptophan-derived metabolites, microbial products, and inflammatory mediators. These signals may influence pulmonary vascular endothelial and smooth muscle cell phenotypes and contribute to pulmonary arteriolar wall thickening and luminal narrowing. In the opposite direction, pulmonary hypertension-associated hypoxia, intestinal hypoperfusion, venous congestion, and systemic inflammation may impair intestinal barrier function and alter the gut microbial environment. Together, these processes may form a bidirectional gut–lung interaction involved in pulmonary vascular remodeling.

## 2 Pulmonary vascular remodeling in pulmonary hypertension

Pulmonary vascular remodeling is an important pathological basis for the progression of PH and the sustained increase in pulmonary vascular resistance. Although the etiologies and histological features differ among PH groups, common changes include pulmonary arterial intimal thickening, medial hypertrophy, muscularization of distal arterioles, adventitial fibrosis, and abnormal extracellular matrix deposition; occlusive lesions and *in situ* thrombosis may also occur in some patients with severe PAH (Morrell et al., 2009; Li et al., 2024; Mocumbi et al., 2024). These structural changes thicken the vascular wall, narrow the lumen, and reduce pulmonary vascular compliance, thereby imposing a sustained increase in right ventricular afterload and ultimately leading to right heart failure. Pulmonary vascular remodeling is therefore not simply a consequence of sustained vasoconstriction, but a dynamic pathological process involving pulmonary arterial endothelial cells, pulmonary arterial smooth muscle cells, fibroblasts, immune cells, and the extracellular matrix (Hong et al., 2021; Mocumbi et al., 2024). The main cellular and pathological processes involved in pulmonary vascular remodeling are shown in **Figure 2**.

**Figure 2:**
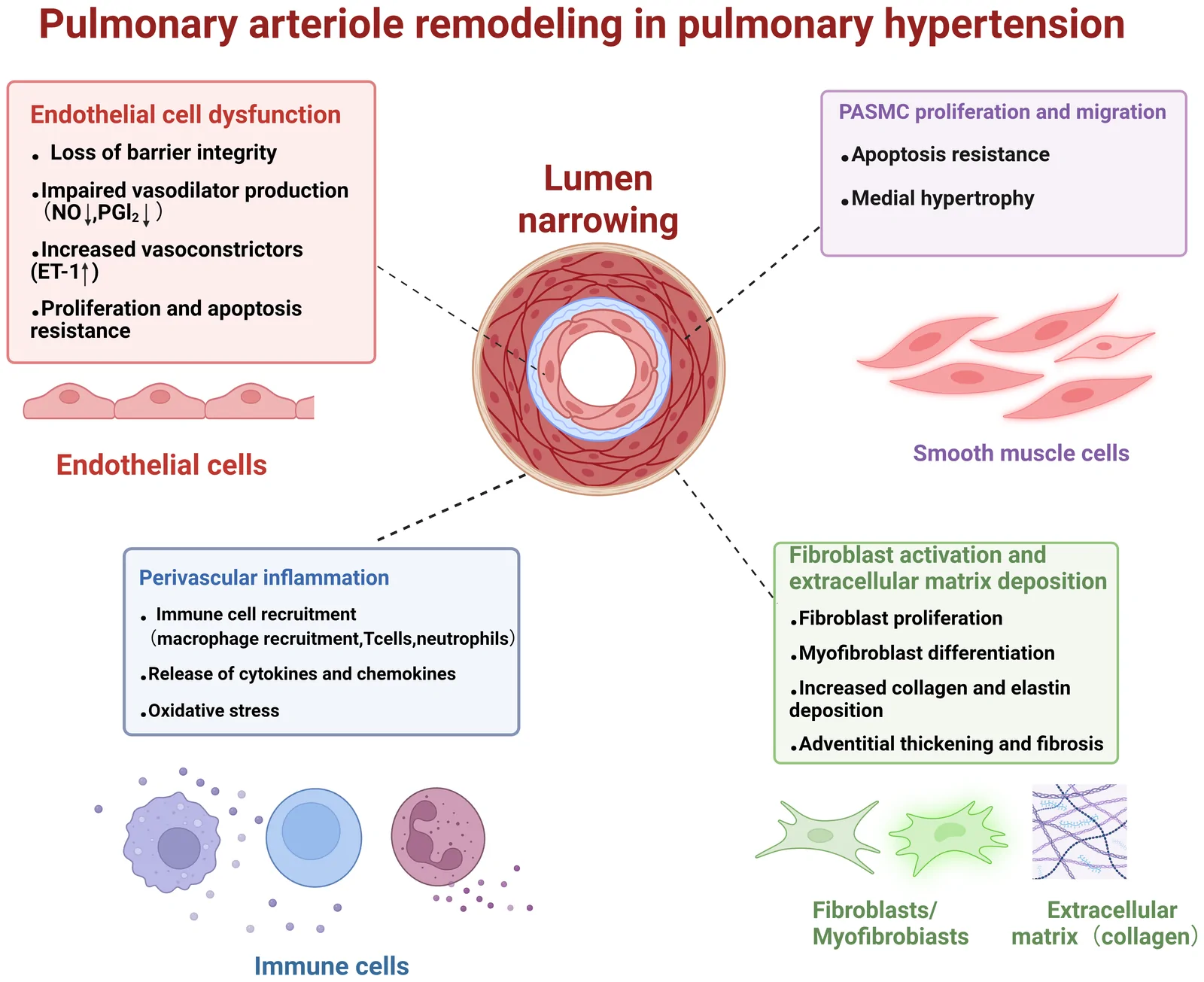
Cellular and structural basis of pulmonary arteriolar remodeling in pulmonary hypertension. Pulmonary arteriolar remodeling results from coordinated alterations in vascular and perivascular cell populations. Endothelial cell dysfunction is characterized by impaired barrier integrity, reduced production of vasodilators, increased vasoconstrictor signaling, and increased proliferation and apoptosis resistance. Pulmonary arterial smooth muscle cell proliferation, migration, and apoptosis resistance contribute to medial hypertrophy. The recruitment of macrophages, T cells, and neutrophils, together with the release of cytokines, chemokines, and reactive oxygen species, promotes perivascular inflammation. Fibroblast proliferation, myofibroblast differentiation, and extracellular matrix deposition lead to adventitial thickening and fibrosis. These processes cause pulmonary arteriolar wall thickening and luminal narrowing, thereby increasing pulmonary vascular resistance. NO, nitric oxide; PGI_2_, prostacyclin; ET-1, endothelin-1; PASMC, pulmonary arterial smooth muscle cell; ↑, increased; ↓, decreased.

Pulmonary arterial endothelial cell dysfunction is an important component of the development and progression of pulmonary vascular remodeling. Under physiological conditions, endothelial cells maintain pulmonary vascular homeostasis by releasing vasodilatory and antiproliferative mediators, including nitric oxide and prostacyclin, while preserving vascular barrier integrity and anti-inflammatory and antithrombotic states. In response to hypoxia, inflammation, mechanical stress, and metabolic disturbance, endothelial vasodilatory and antiproliferative functions decline, whereas vasoconstrictor, proinflammatory, and pro-proliferative signaling increases, accompanied by disruption of intercellular junctions and increased vascular permeability. VEGF-A/VEGFR2 Y949 signaling-mediated vascular permeability promotes macrophage infiltration and hypoxia-induced pulmonary vascular remodeling (Zhou et al., 2022). Endothelial cells in PH also show marked heterogeneity: some microvascular endothelial cells undergo apoptosis, whereas other endothelial subpopulations acquire apoptosis-resistant and abnormal reparative or proliferative phenotypes and contribute to distal arteriole muscularization (Zhang et al., 2024). Recent studies have further implicated altered ErbB3/HIF signaling and endothelial metabolic reprogramming in endothelial dysfunction and PH progression (Bian et al., 2024; Luo et al., 2024). These endothelial phenotypic and metabolic abnormalities provide an important cellular context for understanding the effects of tryptophan-derived metabolites on the pulmonary vasculature.

Abnormal proliferation, migration, and apoptosis resistance of pulmonary arterial smooth muscle cells (PASMCs) constitute the core cellular basis of pulmonary arterial medial thickening and distal arteriole muscularization. In response to hypoxia, inflammatory mediators, growth factors, and metabolic abnormalities, PASMCs can shift from a contractile phenotype to proliferative, migratory, and synthetic phenotypes (Morrell et al., 2009; Mocumbi et al., 2024). AQP1 deletion attenuates hypoxia-induced increases in pulmonary arterial pressure, right ventricular hypertrophy, and pulmonary vascular remodeling in mice, suggesting that AQP1-mediated PASMC migration and proliferation may contribute to the development and progression of hypoxic PH (Liu et al., 2019). Platelet-derived growth factor, 5-HT, endothelin-1, interleukin-6, and oxidative stress can activate pathways that promote proliferation and matrix synthesis, with calpain-2 serving as one downstream node shared by multiple remodeling mediators (Kovacs et al., 2016). Osteoprotegerin and syndecan-4 are involved in PASMC proliferation and migration and in endothelial–smooth muscle cell communication, respectively (Lawrie et al., 2008; Wang et al., 2024), whereas smooth muscle cell-specific ADAR1 deficiency aggravates vascular remodeling and the PH phenotype (Kim et al., 2026). Among these mediators, 5-HT is particularly relevant to the present review because it directly links tryptophan metabolism to pulmonary vasoconstriction and PASMC proliferation.

Pulmonary vascular remodeling is also influenced by perivascular immune and inflammatory responses, fibroblast activation, and extracellular matrix remodeling. Neutrophils, macrophages, and lymphocytes can accumulate around pulmonary vessels, where they release cytokines, chemokines, and reactive oxygen species that exert sustained effects on endothelial cells and PASMCs. Neutrophil-derived S100A9 induces endothelial dysfunction through RAGE/PI3K/AKT signaling and promotes pulmonary vascular wall thickening (Guo et al., 2025). Interactions between lymphocytes and macrophages can further promote inflammatory responses and PASMC proliferation during pulmonary vascular remodeling (Li et al., 2024). Meanwhile, activated fibroblasts and myofibroblasts increase the synthesis of collagen and other extracellular matrix components, leading to adventitial thickening and increased vascular wall stiffness. The abnormal matrix can in turn maintain the proliferative phenotype of vascular wall cells through mechanical and cell-adhesion signaling, creating a local feedback loop in which inflammation, cell proliferation, and matrix deposition reinforce one another. Single-cell transcriptomic studies further indicate marked phenotypic heterogeneity and altered intercellular communication among endothelial cells, smooth muscle cells, fibroblasts, and immune cells in different PH models (Hong et al., 2021). These immune and inflammatory processes also provide an important context for the subsequent discussion of the interactions between immune regulation and tryptophan metabolism. Pulmonary vascular remodeling is therefore not driven independently by a single cell type or signaling pathway, but is a complex process jointly driven by endothelial dysfunction, abnormal PASMC proliferation, immune and inflammatory responses, oxidative stress, metabolic reprogramming, and extracellular matrix deposition (Hong et al., 2021; Mocumbi et al., 2024; Olajuyin et al., 2024).

## 3 The gut–lung axis in pulmonary hypertension

The gut–lung axis refers to a bidirectional communication network between the gut microbiota and the lung mediated by circulating, immune, and metabolic signals. Early work by Pugin and Chevrolet described an intestine–liver–lung axis in septic syndrome (Pugin and Chevrolet, 1991). The gut microbiota comprises bacteria, fungi, archaea, viruses, and bacteriophages, and its composition and function are influenced by diet, age, living environment, medications, and host disease. Under physiological conditions, the gut microbiota participates in nutrient metabolism, immune system development, and the maintenance of mucosal homeostasis; sustained alterations in community structure, functional potential, and metabolic activity may manifest as gut microbial dysbiosis. Advances in 16S rRNA sequencing, metagenomics, and metabolomics have increasingly identified varying degrees of intestinal microbial disturbance in patients with PH and animal models, as summarized in **Table 1**. These findings suggest that the gut microbiota and its metabolites may contribute to the immune and metabolic changes associated with PH and may have potential diagnostic and therapeutic value (Yang et al., 2025; Qiu et al., 2026).

**Table 1:** Representative clinical and experimental studies of gut microbiota and metabolite alterations in pulmonary hypertension.

Study population or model	Main gut microbial changes	Main metabolic changes	Evidence category	Reference
Patients with PAH	↓ α-diversity and richness ↑ *Bifidobacterium* ↓ *Coprococcus*, *Butyrivibrio*, *Eubacterium*, *Akkermansia*, *Bacteroides*, and Lachnospiraceae	↓ Functions associated with SCFA production; ↑ Potential for TMA/TMAO production	Clinical association	(Kim et al., 2020)
Patients with PAH	↓ Microbial richness and diversity ↑ *Streptococcus salivarius* ↓ *Butyrivibrio* sp. CAG 318, *Bifidobacterium angulatum*, and *Eubacterium rectale*	↓ SCFAs	Clinical association	(Moutsoglou et al., 2023)
Rats with chronic hypoxia-induced PH	↑ Peptostreptococcaceae, unclassified Clostridiales, and *Romboutsia* ↓ Bacteroidaceae and Prevotellaceae	Altered TMAO-related metabolism	Preclinical evidence	(Luo et al., 2023)
Rats with SU5416/hypoxia-induced PH	↑ Ruminococcaceae and Peptostreptococcaceae ↓ Lactobacillaceae and Rikenellaceae	–	Preclinical evidence	(Luo et al., 2023)
Rats with MCT-induced PH	↑ Firmicutes, Actinobacteria, *Turicibacter*, and Erysipelotrichaceae ↓ Bacteroidetes and Verrucomicrobia	–	Preclinical evidence	(Luo et al., 2023)
Rats with chronic hypoxia-induced PH	↑ Firmicutes/Bacteroidetes ratio ↑ Erysipelotrichaceae, Peptostreptococcaceae, and Helicobacteraceae ↓ Muribaculaceae, Ruminococcaceae, Christensenellaceae, and *Eubacterium coprostanoligenes* group	↑ Trp and indole-related metabolites; ↓ Kyn-related metabolites	Preclinical evidence	(Shuai et al., 2025)
Rats with chronic hypoxia-induced PH	↑ *Candidatus Saccharimonas* and Campilobacterota ↓ *Blautia*, *Ruminococcus*, *Alloprevotella*, and *Turicibacter* ↓ Bacteroidota, Proteobacteria, and Chloroflexi	↓ SCFAs	Preclinical evidence	(Cao et al., 2024)
Rats with MCT-induced PAH	↓ Microbial diversity ↑ Firmicutes, Proteobacteria, and Actinobacteria ↑ *Allobaculum*, *Ralstonia*, and *Bifidobacterium* ↓ Bacteroidota and Spirochaetota ↓ *Lactobacillus* and *Romboutsia*	Abnormalities in multiple microbiota-related fecal metabolites	Preclinical evidence	(Hong et al., 2021)
Rats with SU5416/hypoxia-induced PAH	↑ Firmicutes/Bacteroidetes ratio ↑ Lachnospiraceae NK4A136 group, *Bifidobacterium*, *Allobaculum*, *Parasutterella*, and Prevotellaceae UCG-001 ↓ *Akkermansia*, *Bacteroides*, *Enterococcus*, and *Dehalobacterium*	–	Preclinical evidence	(Sanada et al., 2020)
Rats with MCT-induced PAH	↓ *Lactobacillus* Altered overall bacterial and fungal community structure	–	Preclinical evidence	(Prisco et al., 2025)
Rats with chronic hypoxia-induced PH	↓ α-diversity and microbial richness ↑ Firmicutes/Bacteroidetes ratio ↑ *Escherichia–Shigella* and *Helicobacter* ↓ *Lactobacillus*, *Lactobacillus johnsonii*, and *Prevotella*	↑ 5-HT	Preclinical evidence	(Wang et al., 2025)
Sprague–Dawley rats exposed to hypobaric hypoxia	↑ Lachnospiraceae and Prevotellaceae	↑ SCFAs ↑ Indole-related metabolites	Preclinical evidence	(Chen et al., 2025)

PAH, pulmonary arterial hypertension; PH, pulmonary hypertension; MCT, monocrotaline; SCFAs, short-chain fatty acids; TMAO, trimethylamine N-oxide; Trp, tryptophan; Kyn, kynurenine; ↑, increased; ↓, decreased.

### 3.1 Gut microbial dysbiosis in pulmonary hypertension

An increasing number of studies have documented varying degrees of gut microbial dysbiosis in patients with PH and related animal models. In PAH, microbial composition and functional gene profiles differ from those of healthy individuals: some taxa associated with butyrate or propionate production are reduced, whereas functional capacities related to amino acid biosynthesis, trimethylamine production, and purine metabolism are increased (Kim et al., 2020). Multi-omics studies have further shown lower microbial richness and diversity, reductions in taxa or functions associated with short-chain fatty acid (SCFA) and secondary bile acid production, and changes in circulating microbiota-related metabolites; some of these features are associated with mean pulmonary arterial pressure, pulmonary vascular resistance, and pulmonary arterial compliance (Moutsoglou et al., 2023). Multi-kingdom microbiome studies also show concurrent changes in bacterial, fungal, and archaeal communities (Chen et al., 2025). Changes in the gut microbiota and metabolite profiles have been observed in chronic hypoxia (Luo et al., 2023; Cao et al., 2024; Shuai et al., 2025), SU5416/hypoxia (Callejo et al., 2018; Luo et al., 2023), monocrotaline (Hong et al., 2021; Luo et al., 2023), and left pulmonary artery ligation models (Chen et al., 2022). Some chronic hypoxia and SU5416/hypoxia models show an increased Firmicutes/Bacteroidetes ratio and reductions in Lactobacillaceae or SCFA-producing taxa, although these patterns have not been reproduced in all models. Direct comparisons among PH models also show that microbial diversity, dominant taxa, and metabolic functions vary with the disease trigger, disease severity, and disease stage (Luo et al., 2023). Microbiota from high-altitude animal donors can also alter pulmonary gene expression and metabolic adaptation in rats exposed to hypobaric hypoxia, accompanied by changes in metabolites such as indole-3-lactic acid (Chen et al., 2025). Overall, studies have not reached consistent conclusions regarding differential genera or the direction of metabolic changes, and stable associations with PH severity and prognosis require further validation.

### 3.2 Intestinal barrier dysfunction and microbial translocation

The intestinal mucosal barrier consists of the mucus layer, epithelial cells, intercellular tight junctions, and the mucosal immune system and serves as a key defense against the entry of intestinal microorganisms and their products into the circulation. During PH progression, reduced cardiac output, mesenteric hypoperfusion, venous congestion, and intestinal wall edema may reduce the oxygen and energy supply to epithelial cells and disrupt intercellular junctions; hypoxia, oxidative stress, and systemic inflammation can further aggravate epithelial injury (Yang et al., 2025; Qiu et al., 2026). Intestinal barrier integrity is essential for maintaining gut microbial homeostasis, whereas dysbiosis can in turn impair barrier function. Clinical evidence suggests altered gut barrier function in PAH, although direct evidence of increased intestinal permeability remains limited (Moutsoglou et al., 2023). Shortened intestinal villi, reduced goblet cell numbers, intestinal wall fibrosis, and altered tight-junction proteins have also been observed in developmental, MCT, and chronic hypoxia-associated PH models (Sharma et al., 2020a; Sharma et al., 2020b; Wedgwood et al., 2020b). These findings suggest that PH may be accompanied by intestinal barrier injury. Once the barrier is disrupted and intestinal permeability increases, microbial components such as LPS released by Gram-negative bacteria, peptidoglycan, and bacterial DNA can more readily enter the circulation. Through pattern-recognition receptors such as Toll-like receptors, these components may activate immune cells, pulmonary vascular endothelial cells, and PASMCs, promote the release of IL-6, TNF-α, and chemokines, and thereby aggravate perivascular inflammation and pulmonary vascular remodeling (Thenappan et al., 2019; Wedgwood et al., 2020a). Developmental models suggest that TLR4-related inflammation contributes to pulmonary vascular abnormalities (Wedgwood et al., 2020a), whereas studies of chronic hypoxic PH show that some interventions improve intestinal barrier and inflammatory status while also improving the PH phenotype (Sharma et al., 2020b; Oliveira et al., 2022; Wang et al., 2025).

### 3.3 Microbial metabolites and systemic inflammatory signaling

In addition to structural microbial components such as LPS, peptidoglycan, and bacterial nucleic acids, metabolites derived from the gut microbiota are important signaling molecules in gut–lung communication. These metabolites can regulate epithelial barrier function and mucosal immunity locally in the intestine and may also enter the systemic circulation after absorption, thereby affecting the pulmonary vasculature and other distant tissues. Microbiota-related metabolites that have received particular attention in PH include short-chain fatty acids (SCFAs), trimethylamine N-oxide (TMAO), secondary bile acids, and tryptophan-related metabolites (Yang et al., 2025; Qiu et al., 2026). SCFAs help maintain the energy supply of intestinal epithelial cells, tight-junction integrity, and immune tolerance; in rats with hypoxic PH, butyrate supplementation attenuated pulmonary vascular inflammation and remodeling and reduced right ventricular hypertrophy (Karoor et al., 2021). By contrast, elevated circulating TMAO levels are associated with greater PAH severity and poorer prognosis (Yang et al., 2022). Animal and cell studies further suggest that TMAO may contribute to disease progression by promoting inflammation, impairing pulmonary vascular endothelial function, and enhancing PASMC proliferation (Huang et al., 2022). Secondary bile acids and other microbiota-related metabolites may also regulate immune responses, energy metabolism, and vascular function through specific receptor pathways, although direct evidence in PH remains limited (Yang et al., 2025; Qiu et al., 2026).

The pathological significance of PH-associated gut microbial dysbiosis is not limited to changes in community composition, but may also involve remodeling of microbial metabolic function and its effects on systemic inflammation and vascular homeostasis (Yang et al., 2025; Qiu et al., 2026). Circulating metabolite levels, however, are influenced by dietary intake, microbial production, intestinal absorption, host conversion, tissue uptake and utilization, and hepatic and renal clearance. Changes in microbial composition or circulating metabolite levels alone cannot determine the origin of a metabolite or its direct effect on the pulmonary vasculature (Yang et al., 2025; Qiu et al., 2026). Most available studies report parallel changes in the microbiota, metabolites, and PH phenotype and do not establish a causal chain among them; studies that trace a specific metabolite to its source and verify its pulmonary vascular effects remain limited (Callejo et al., 2018; Hong et al., 2021; Chen et al., 2022; Luo et al., 2023; Cao et al., 2024). Among these metabolic signals, tryptophan is of particular interest because its metabolism is regulated by diet, host immunity, tissue enzyme activity, and the gut microbiota and generates several classes of bioactive molecules that can act on barrier function, inflammation, and vascular function (Agus et al., 2018; Roager and Licht, 2018). Tryptophan metabolism may therefore constitute an important metabolic link through which intestinal dysbiosis affects pulmonary vascular remodeling. The main gut microbial and metabolic changes reported in patients with PH and animal models are summarized in **Table 1**.

## 4 Tryptophan metabolism at the host–gut microbiota interface

Tryptophan is an essential aromatic amino acid that humans cannot synthesize and must obtain mainly from the diet. In addition to its role in protein synthesis, tryptophan gives rise to numerous bioactive metabolites through host and microbial pathways. After digestion in the gastrointestinal tract and absorption in the small intestine, dietary tryptophan enters the portal circulation and is then taken up by the liver, immune cells, intestinal mucosa, and other peripheral tissues; a fraction that reaches the colon is further metabolized by gut microorganisms (Liu et al., 2020; Xue et al., 2023). Tryptophan is metabolized through three major pathways: the kynurenine, serotonin, and indole pathways. The kynurenine pathway accounts for most tryptophan degradation and participates in immune regulation and *de novo* nicotinamide adenine dinucleotide (NAD^+^) synthesis (Cervenka et al., 2017; Kennedy et al., 2017; Xue et al., 2023). The 5-HT pathway is controlled mainly by TPH1 and TPH2 (Mawe and Hoffman, 2013; Spohn and Mawe, 2017; Nonogaki and Kaji, 2025), whereas tryptophan reaching the colon can be converted by different microbial enzyme systems into indole, tryptamine, and several indole acid derivatives (Agus et al., 2018; Roager and Licht, 2018; Ye et al., 2022). The three pathways share tryptophan as a substrate and are jointly influenced by inflammation, diet, and the microbial environment. The principal metabolic routes are shown in **Figure 3**.

**Figure 3:**
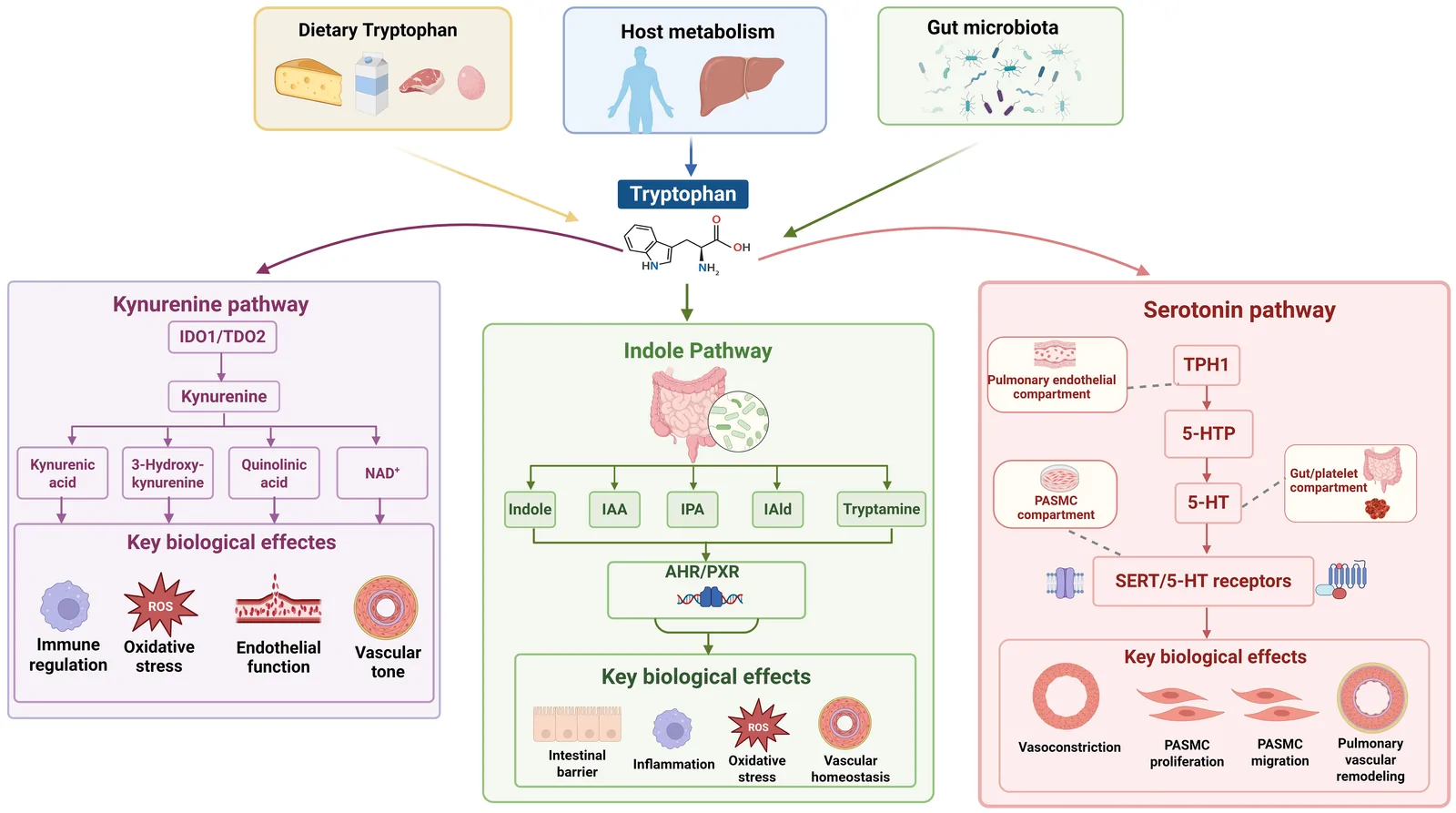
Tryptophan metabolism at the host–gut microbiota interface. Dietary tryptophan enters a shared metabolic pool and is subsequently metabolized through three major pathways. In host tissues, IDO1 and TDO2 initiate the kynurenine pathway, generating Kyn and downstream metabolites, including KYNA, 3-HK, quinolinic acid, and NAD^+^. These metabolites participate in immune regulation, oxidative stress, endothelial function, and vascular tone. In the serotonin pathway, TPH1-dependent 5-HT synthesis occurs in distinct compartments. Gut/platelet 5-HT, pulmonary endothelial TPH1-dependent local 5-HT synthesis, and PASMC SERT/5-HT receptor signaling are therefore shown separately. PASMC SERT/5-HT receptor signaling contributes to vasoconstriction, PASMC proliferation and migration, and pulmonary vascular remodeling. In the intestinal lumen, gut microorganisms convert tryptophan into indole and its derivatives, including IAA, IPA, IAld, and tryptamine. These metabolites may signal through AHR and/or PXR, thereby influencing intestinal barrier integrity, inflammation, oxidative stress, and vascular homeostasis. Arrows indicate major metabolic conversions and regulatory relationships. AHR, aryl hydrocarbon receptor; 3-HK, 3-hydroxykynurenine; IAA, indole-3-acetic acid; IAld, indole-3-aldehyde; IDO1, indoleamine 2,3-dioxygenase 1; IPA, indole-3-propionic acid; Kyn, kynurenine; KYNA, kynurenic acid; NAD^+^, nicotinamide adenine dinucleotide; PASMC, pulmonary arterial smooth muscle cell; PXR, pregnane X receptor; ROS, reactive oxygen species; SERT, serotonin transporter; TDO2, tryptophan 2,3-dioxygenase 2; TPH1, tryptophan hydroxylase 1; 5-HT, 5-hydroxytryptamine; 5-HTP, 5-hydroxytryptophan.

### 4.1 The kynurenine pathway

The kynurenine pathway is the principal route of host tryptophan degradation. Tryptophan is converted to N-formylkynurenine by tryptophan 2,3-dioxygenase (TDO) or indoleamine 2,3-dioxygenase (IDO) and is then further converted to kynurenine (Kyn) (Cervenka et al., 2017; Kennedy et al., 2017). TDO is expressed mainly in the liver and contributes to systemic tryptophan homeostasis, whereas IDO1 is widely expressed in immune cells, endothelial cells, and other peripheral tissues and can be induced by interferon-γ and other inflammatory signals (Kennedy et al., 2017; Gao et al., 2018). IDO2 also catalyzes the conversion of tryptophan to Kyn, but its tissue distribution, catalytic activity, and physiological functions differ from those of IDO1. Infection, chronic inflammation, and immune activation may therefore alter the allocation of tryptophan to the kynurenine pathway. Kyn is a major branch point in this pathway: it can be converted to kynurenic acid (KYNA) by kynurenine aminotransferases or enter the branch leading to 3-hydroxykynurenine, quinolinic acid, and *de novo* NAD^+^ synthesis through kynurenine 3-monooxygenase (KMO) (Cervenka et al., 2017; Kennedy et al., 2017). In addition to influencing metabolite allocation, KMO can regulate mitochondrial dynamics, although this mechanism has been demonstrated mainly in non-PH models (Maddison et al., 2020). Downstream metabolites differ in receptor activity, redox properties, and immune effects. Abnormal NAD metabolism in PH is not confined to kynurenine-dependent *de novo* synthesis; the NAMPT-mediated NAD salvage pathway can also promote pulmonary vascular remodeling (Chen et al., 2017). Although the kynurenine pathway is primarily a host metabolic pathway, the gut microbiota may indirectly regulate IDO1/TDO activity by competing for tryptophan, altering the intestinal barrier, and affecting inflammatory mediators (Kennedy et al., 2017; Agus et al., 2018). Kyn and some downstream products can act on metabolic sensing pathways such as AHR, whereas KYNA can activate GPR35, thereby influencing immune tolerance and cellular metabolism (Agus et al., 2018; Gao et al., 2018). These features make the kynurenine pathway an important link between the intestinal microenvironment, host inflammation, and metabolic homeostasis.

### 4.2 The serotonin pathway

Only a small fraction of systemic tryptophan enters the serotonin pathway, but the resulting 5-HT has broad physiological effects. Tryptophan hydroxylase (TPH) first converts tryptophan to 5-hydroxytryptophan (5-HTP), which is then converted to 5-HT by aromatic L-amino acid decarboxylase (Mawe and Hoffman, 2013; Spohn and Mawe, 2017). TPH has two main isoforms: TPH1 is expressed mainly in peripheral tissues, particularly enterochromaffin cells, and is the key rate-limiting enzyme in peripheral 5-HT synthesis, whereas TPH2 is expressed mainly in serotonergic neurons of the central and enteric nervous systems. Changes in TPH1 may also affect the allocation of tryptophan to metabolites other than 5-HT (Nonogaki and Kaji, 2025). Most of the body’s 5-HT is synthesized by gastrointestinal enterochromaffin cells. Once released, gut-derived 5-HT regulates gastrointestinal motility, secretion, and sensation locally and can also enter the circulation, where it is taken up and stored by platelets through the serotonin transporter (SERT) (Mawe and Hoffman, 2013; Spohn and Mawe, 2017). Free 5-HT can be further metabolized by monoamine oxidase to 5-hydroxyindoleacetic acid. Because peripheral 5-HT generally does not cross the blood–brain barrier, the peripheral and central 5-HT systems are relatively independent in their origins and ranges of action (Spohn and Mawe, 2017; Agus et al., 2018). Through different receptor subtypes, 5-HT also regulates gastrointestinal function, platelet activation, immune responses, and vascular function, with its effects depending on receptor subtype, target cell, and local concentration (Mawe and Hoffman, 2013; Spohn and Mawe, 2017).

The gut microbiota is not a major direct source of host 5-HT, but it can influence peripheral 5-HT synthesis by regulating enterochromaffin cell function. Germ-free animals have lower colonic and circulating 5-HT levels, whereas colonization with selected spore-forming bacteria can partially restore these levels; microbial metabolites implicated in this effect include SCFAs and secondary bile acids (Yano et al., 2015). SCFAs can directly promote TPH1 expression and 5-HT production in enterochromaffin cells (Reigstad et al., 2015). In a model of multiple sclerosis, microbiota-derived indole-3-aldehyde regulated immune responses and helped maintain immune homeostasis through AHR–TPH1-related signaling (Zelante et al., 2024). These findings suggest that the gut microbiota and its metabolites may influence host immune homeostasis by altering the allocation of tryptophan between the 5-HT and kynurenine pathways.

However, these findings mainly demonstrate microbiota-dependent regulation of intestinal 5-HT production outside the PH setting. This intestinal source of 5-HT should be distinguished from pulmonary endothelial TPH1-dependent 5-HT synthesis, and a direct causal link between microbiota-regulated enterochromaffin 5-HT production and pulmonary vascular remodeling in PH has not yet been established.

### 4.3 The indole pathway

The microbial indole pathway is an important branch of gut microbial tryptophan metabolism. Gut microorganisms convert tryptophan to indole through tryptophanase (TnaA) or to tryptamine through tryptophan decarboxylase (TDC); different microbial taxa can further convert tryptophan through diverse enzyme systems into derivatives such as indole-3-propionic acid (IPA), indole-3-acetic acid (IAA), indole-3-lactic acid (ILA), indole-3-aldehyde (IAld), and indole-3-acrylic acid (IA) (Dodd et al., 2017; Ye et al., 2022). Dietary fiber and metabolic interactions among gut microorganisms can redirect the allocation of tryptophan among different indole products (Sinha et al., 2024). Product profiles differ among microbial species; for example, *Lactobacillus* species can produce ILA or IAld (Zelante et al., 2013; Cervantes-Barragan et al., 2017), whereas some *Clostridium* and *Peptostreptococcus* species contribute to the production of IPA or IA (Dodd et al., 2017; Wlodarska et al., 2017). These metabolites can act locally in the intestine and can also enter the circulation after absorption. Some indole metabolites maintain mucosal homeostasis by regulating IL-22, the Th17/Treg balance, the mucus layer, and tight junctions through AHR or PXR (Zelante et al., 2013; Cervantes-Barragan et al., 2017; Wlodarska et al., 2017; Ye et al., 2022).

## 5 Tryptophan metabolism in pulmonary vascular remodeling in pulmonary hypertension

### 5.1 The kynurenine pathway and pulmonary hypertension

Clinical studies indicate marked dysregulation of kynurenine metabolism in patients with PAH. Several studies have reported lower tryptophan (Trp) levels and higher Kyn levels or Kyn/Trp ratios in PAH, with kynurenine pathway activity associated with immune activation, pulmonary vascular resistance, functional status, and survival (Jasiewicz et al., 2016; Nagy et al., 2017; Cai et al., 2022; Simpson et al., 2023). In individuals with systemic sclerosis at high risk of PAH, related metabolic abnormalities can precede the diagnosis of PAH and change dynamically as the disease progresses (Simpson et al., 2023). However, the direction of change in Kyn is not consistent across cohorts. A small study found lower Kyn levels in patients with PAH than in controls, although within the PAH group Kyn remained associated with disease-related indices such as poly(ADP-ribose) polymerase 1 (PARP-1) and miR-210 (Akgun et al., 2022). This finding suggests that kynurenine metabolic profiles may be influenced by disease subtype, disease stage, and comorbidities. Metabolites of the kynurenine pathway may also have value as biomarkers. Combined measurement of IDO-dependent metabolites and endothelial cell-specific molecule 1 (endocan) may aid the diagnosis of PH during pregnancy (Peng et al., 2025), and a targeted metabolomic study of idiopathic PAH identified Kyn and Trp as candidate biomarkers, although the sample size was small (Yang et al., 2024). Transpulmonary metabolomic analysis showed exercise-stage-specific differences in the handling of Kyn and other tryptophan catabolites in connective tissue disease-associated pulmonary vascular disease, rather than a simple change in peripheral concentrations (Lee et al., 2025).

The pulmonary vascular effects of the kynurenine pathway are highly context dependent. Kyn increases intracellular cyclic adenosine monophosphate and cyclic guanosine monophosphate levels in PASMCs and relaxes isolated pulmonary arteries, suggesting that increased Kyn may exert a compensatory effect when pulmonary vascular tone is elevated (Nagy et al., 2017). However, the cellular source of IDO1 in PAH remains uncertain. Nagy et al. reported elevated circulating Kyn without increased IDO expression in lung tissue or pulmonary arteries (Nagy et al., 2017). Similarly, Cai et al. found increased IDO1 expression in peripheral blood mononuclear cells, but not in lung tissue, in patients with PAH and MCT-PH rats (Cai et al., 2025). *In vitro* experiments further showed that increased IDO1 activity promoted Kyn production, while elevated Kyn enhanced *de novo* NAD^+^ synthesis and pulmonary microvascular endothelial cell proliferation. IDO1 inhibition reversed these effects and, in MCT-PH rats, attenuated pulmonary vascular remodeling, inflammation, and right ventricular remodeling (Cai et al., 2025). Thus, current evidence supports a potential contribution of IDO1-dependent kynurenine metabolism to pulmonary vascular remodeling but does not establish disease-associated *in vivo* upregulation of endothelial IDO1. The relative contributions of circulating immune cells and pulmonary vascular cells therefore remain unresolved. IDO1-mediated tryptophan depletion and the accumulation of metabolites such as Kyn can also regulate T-cell activation, differentiation, and immune tolerance, thereby affecting local pulmonary vascular immune homeostasis (Liu et al., 2010; Zhai et al., 2015). Accordingly, the IDO1–Kyn pathway remains a potential therapeutic target, but the relevant cellular source and the most appropriate compartment for therapeutic intervention require further clarification.

### 5.2 The role of the serotonin pathway in pulmonary vascular remodeling in pulmonary hypertension

5-HT functions both as a pulmonary vasoconstrictor and as a growth factor for PASMCs (Eddahibi et al., 2001; Marcos et al., 2004; MacLean, 2018). Studies using isolated human small muscular pulmonary arteries show that the 5-HT_1_B receptor plays an important role in 5-HT-induced contraction (Morecroft et al., 1999). Evidence from 5-HT_1_B antagonism, SERT inhibition, and their combination supports a functional interaction between the receptor and transporter in pulmonary arteries (Keegan et al., 2001; Morecroft et al., 2005; Baranowska-Kuczko et al., 2020). In PASMCs derived from patients with idiopathic PAH, SERT expression and 5-HT uptake are increased, and the proliferative response to 5-HT is more pronounced. SERT can mediate the entry of 5-HT into PASMCs and act in concert with 5-HT_1_B receptor signaling to activate intracellular pro-proliferative pathways (Eddahibi et al., 2001; Marcos et al., 2004; Eddahibi et al., 2006). Cases of drug- and toxin-associated PAH also indicate that the long-term safety of interventions targeting 5-HT requires careful consideration (Montani et al., 2013).

5-HT can also alter pulmonary vascular cell phenotypes through oxidative stress and Ca²^+^ signaling. Studies in rats with chronic hypoxic PH and in PASMCs show that 5-HT promotes cell proliferation and migration through NOX4-dependent reactive oxygen species production and TRPM2-mediated Ca²^+^ influx (Song et al., 2021). Extracellular superoxide enhances 5-HT-induced pulmonary arterial contraction (Liu and Folz, 2004), whereas 5-HT_1_B–NOX1 signaling is associated with oxidative stress and proliferation in pulmonary vascular cells from patients with PAH (Hood et al., 2017). The 5-HT_2_A receptor and SERT also promote pulmonary fibroblast proliferation (Welsh et al., 2004). In *Bmpr2*-heterozygous mice, 5-HT stimulation caused a greater rise in pulmonary arterial pressure and more severe pulmonary vascular remodeling (Long et al., 2006). Deficiency of *Tph1* or SERT attenuates hypoxic PH, whereas smooth muscle cell-specific SERT overexpression induces PH (Eddahibi et al., 2000; Guignabert et al., 2006; Izikki et al., 2007; Morecroft et al., 2007). Targeted knockdown of *Tph1* in pulmonary arterial endothelial cells likewise attenuates hypoxic PH and pulmonary vascular remodeling (Morecroft et al., 2012). Separately, 5-HT_2_B receptors on bone marrow-derived cells contribute to pulmonary vascular disease (Launay et al., 2012). Pharmacological TPH1 inhibition attenuates pulmonary vascular remodeling in MCT and SU5416/hypoxia rats and produces additive effects with an endothelin receptor antagonist (Aiello et al., 2017). The inhaled TPH1 inhibitor TPT-004 also improves hemodynamics and remodeling in established experimental PAH (Legchenko et al., 2025). These pulmonary vascular and hematopoietic mechanisms should be distinguished from microbiota-mediated regulation of enterochromaffin TPH1 and intestinal 5-HT production. A direct causal link between microbiota-regulated intestinal 5-HT production and pulmonary vascular remodeling in PH has not yet been established. In the randomized phase 2b ELEVATE-2 trial, however, systemic peripheral TPH1 inhibition with rodatristat ethyl reduced indices of 5-HT synthesis but markedly worsened PVR: the least-squares mean percentage change from baseline to week 24 was approximately 5.8% with placebo versus 63.1% and 64.2% with rodatristat ethyl 300 and 600 mg twice daily, respectively, accompanied by adverse effects on pulmonary hemodynamics and cardiac function (Sitbon et al., 2024).

### 5.3 Potential role of microbiota-related indole metabolism in pulmonary vascular remodeling in pulmonary hypertension

Microbial indole metabolism may represent a potential metabolic link between the gut microbiota and pulmonary vascular remodeling. The gut microbiota converts tryptophan into indole derivatives, some of which modulate inflammation and immune tolerance by inhibiting TNF-α-mediated NF-κB signaling or activating the aryl hydrocarbon receptor (AHR) (Liu et al., 2020; Ye et al., 2022). A gut metagenomic study in patients with PAH identified alterations in microbial pathways related to tryptophan biosynthesis and metabolism (Kim et al., 2020). Abnormalities in tryptophan-related metabolites, including indole-3-acetic acid, have also been observed in rats with chronic hypoxic PH (Cao et al., 2024), although the directions of microbial and metabolic changes differ among models (Luo et al., 2023; Cao et al., 2024). AHR may be an important signaling node linking altered tryptophan metabolism to pulmonary vascular disease. In patients with PAH, serum AHR agonistic activity was associated with poor outcomes; combining an AHR ligand with hypoxia promoted pulmonary vascular lesions, whereas *Ahr* deletion attenuated the phenotype (Masaki et al., 2021). In the SU5416/hypoxia model, the AHR antagonist CH223191 improved hemodynamics and pulmonary vascular remodeling (Dean et al., 2018). However, because AHR responds to microbiota-derived indoles, host-derived Kyn, dietary compounds, and environmental chemicals, and because SU5416 itself has AHR activity, these studies do not establish that AHR effects in PH are driven mainly by gut-derived indoles (Kwapiszewska et al., 2019; Huang et al., 2022). The endogenous AHR ligand ITE was shown to promote transdifferentiation of human pulmonary arterial endothelial cells under hypoxic conditions (Wang et al., 2019).

A recent study showed that IPA inhibited PASMC proliferation, lowered pulmonary arterial pressure and pulmonary vascular resistance, and attenuated pulmonary arterial remodeling in rats with doxorubicin-induced PH associated with left heart disease (PH-LHD); these effects were associated with the Adora1–Akt–mTOR signaling pathway (Wu et al., 2026). Other cardiopulmonary disease models provide mechanistic support for the effects of indole metabolites on distant organs. In a model of heart failure with preserved ejection fraction, IPA improved cardiac diastolic function and metabolic remodeling, reduced oxidative stress and inflammation, and regulated AHR-related NAD^+^ metabolism (Wang et al., 2024). In a model of acute lung injury, microbiota-derived oxindole acted on pulmonary macrophages and suppressed pulmonary inflammation through AHR–RelA–CXCL13 signaling (Tang et al., 2026). These studies suggest that microbiota-derived indole metabolites may influence cardiopulmonary tissue remodeling by regulating cell proliferation, metabolic homeostasis, and local immunity. However, the role of microbiota-derived indole metabolites in PH remains uncertain. Further studies are needed to determine whether they causally link gut dysbiosis to pulmonary vascular remodeling and to identify their relevant target cells and receptors.

Current evidence suggests that the kynurenine, 5-HT, and microbiota-related indole pathways may participate in pulmonary vascular remodeling by regulating pulmonary vascular tone, endothelial homeostasis, and PASMC phenotype; however, the evidence base and degree of clinical translation differ among these pathways. The principal findings are summarized in **Table 2**.

**Table 2:** Evidence linking tryptophan metabolism to pulmonary vascular remodeling in pulmonary hypertension.

Pathway/target	Study population or model	Key metabolites, enzymes, or targets	Main findings related to pulmonary vascular remodeling	Evidence category	Reference
Kynurenine pathway	Patients with PAH, individuals with systemic sclerosis at high risk of PAH, and patients with connective tissue disease-associated pulmonary vascular disease	Trp, Kyn, and Kyn/Trp ratio	Altered kynurenine metabolism is associated with PVR, functional status, and prognosis, although the direction of change in Kyn is not consistent across cohorts	Clinical association	(Jasiewicz et al., 2016; Nagy et al., 2017; Akgun et al., 2022; Cai et al., 2022; Simpson et al., 2023; Yang et al., 2024; Lee et al., 2025; Peng et al., 2025)
Kynurenine pathway	Samples from patients with PAH, pulmonary microvascular endothelial cells, and MCT-PH rats	IDO1, Kyn, and *de novo* NAD^+^ synthesis	Circulating Kyn and PBMC IDO1 are increased in PAH, whereas pulmonary vascular IDO1 upregulation has not been established. Experimental activation of the IDO1–Kyn pathway promotes *de novo* NAD^+^ synthesis and endothelial proliferation, while IDO1 inhibition attenuates pulmonary vascular remodeling in MCT-PH rats.	Clinical association/Preclinical evidence	(Nagy et al., 2017; Cai et al., 2025)
5-HT pathway	Isolated human small muscular pulmonary arteries, PASMCs from patients with IPAH, chronic hypoxic PH rats, and pulmonary vascular cells	5-HT, 5-HT_1_B receptor, SERT, NOX1/NOX4, and TRPM2	5-HT promotes pulmonary vasoconstriction and, through SERT, oxidative stress, and Ca²^+^ signaling, promotes PASMC proliferation and migration and pulmonary fibroblast proliferation	Preclinical evidence	(Morecroft et al., 1999; Eddahibi et al., 2001; Liu and Folz, 2004; Marcos et al., 2004; Welsh et al., 2004; Morecroft et al., 2005; Eddahibi et al., 2006; Long et al., 2006; Hood et al., 2017; MacLean, 2018; Baranowska-Kuczko et al., 2020; Song et al., 2021)
5-HT pathway	*Tph1*- or SERT-modified mice, models involving 5-HT_2_B receptors on bone marrow-derived cells, MCT and SuHx rats, and experimental models treated with an inhaled TPH1 inhibitor	TPH1, SERT, 5-HT_1_B/5-HT_2_B receptors, and TPT-004	*Tph1*/SERT deficiency and pulmonary endothelial Tph1 knockdown attenuate PH, whereas smooth muscle SERT overexpression and bone marrow-derived cell 5-HT_2_B signaling promote disease. TPH1 inhibition improves pulmonary vascular remodeling in experimental PH.	Preclinical evidence	(Eddahibi et al., 2000; Keegan et al., 2001; Guignabert et al., 2006; Izikki et al., 2007; Morecroft et al., 2007; Launay et al., 2012; Morecroft et al., 2012; Aiello et al., 2017; Legchenko et al., 2025)
5-HT pathway	Patients with PAH in the randomized phase 2b ELEVATE-2 trial	Rodatristat ethyl and TPH1	Rodatristat ethyl reduced indices of 5-HT synthesis but markedly worsened PVR and adversely affected pulmonary hemodynamics and cardiac function.	Negative clinical trial	(Sitbon et al., 2024)
Microbiota-related indole pathway/AHR signaling	Serum from patients with PAH, models of AHR deficiency or activation, SuHx rats, and hypoxia-treated human pulmonary arterial endothelial cells	AHR, FICZ, CH223191, and ITE	AHR activation promotes pulmonary vascular disease and endothelial phenotypic transition, whereas *Ahr* deficiency or AHR antagonism attenuates PH and pulmonary vascular remodeling; however, the available studies do not establish that the relevant AHR ligands are derived primarily from the gut microbiota	Clinical association/Preclinical evidence	(Dean et al., 2018; Kwapiszewska et al., 2019; Wang et al., 2019; Masaki et al., 2021; Huang et al., 2022)
Microbiota-related indole pathway	Rats with chronic hypoxic PH, rats with doxorubicin-induced PH-LHD, and PASMCs	IAA, IPA, and Adora1–Akt–mTOR	Indole-related metabolites are altered in chronic hypoxic PH; IPA inhibits PASMC proliferation, lowers pulmonary arterial pressure and PVR, and attenuates pulmonary vascular remodeling in PH-LHD, although the direct evidence is currently based mainly on a single animal model	Preclinical evidence	(Shuai et al., 2025; Wu et al., 2026)

PAH, pulmonary arterial hypertension; IPAH, idiopathic pulmonary arterial hypertension; PH-LHD, pulmonary hypertension associated with left heart disease; MCT, monocrotaline; SuHx, SU5416/hypoxia; PASMCs, pulmonary arterial smooth muscle cells; PVR, pulmonary vascular resistance; Trp, tryptophan; Kyn, kynurenine; IDO1, indoleamine 2,3-dioxygenase 1; NAD^+^, nicotinamide adenine dinucleotide; 5-HT, 5-hydroxytryptamine; TPH1, tryptophan hydroxylase 1; SERT, serotonin transporter; NOX, NADPH oxidase; TRPM2, transient receptor potential melastatin 2; AHR, aryl hydrocarbon receptor; FICZ, 6-formylindolo[3,2-b]carbazole; ITE, 2-(1′H-indole-3′-carbonyl)-thiazole-4-carboxylic acid methyl ester; IAA, indole-3-acetic acid; IPA, indole-3-propionic acid; Adora1, adenosine A1 receptor; Akt, protein kinase B; mTOR, mammalian target of rapamycin.

Across the available studies, the evidence linking the three tryptophan metabolic pathways to pulmonary vascular remodeling in PH is uneven. The direct links between the 5-HT pathway and pulmonary vasoconstriction, abnormal PASMC proliferation, and structural remodeling are relatively clear. The kynurenine pathway is supported by substantial clinical metabolic evidence, but its effects depend on the metabolic branch, cellular source, and duration of action. Evidence for microbiota-related indole metabolism comes mainly from microbiome–metabolome associations and AHR studies; the microbial origin of the relevant metabolites and their direct pulmonary vascular effects remain to be determined. The three pathways may also influence one another through the allocation of tryptophan among pathways, inflammatory status, and receptor signaling, providing a basis for integrated evaluation of their mechanistic relationships and translational value.

## 6 Mechanistic integration, therapeutic strategies, and translational perspectives

### 6.1 Potential mechanistic links among the gut microbiota, tryptophan metabolism, and pulmonary vascular remodeling

As PH progresses, reduced cardiac output, intestinal hypoperfusion, venous congestion, tissue hypoxia, and systemic inflammation can impair intestinal epithelial energy metabolism and tight-junction integrity, increase intestinal permeability, and alter the microbial habitat. Clinical studies and multiple PH animal models show that PH can be accompanied by changes in gut microbial composition, microbial functional gene profiles, and circulating metabolite profiles, although the specific patterns differ among disease subtypes and experimental models (Callejo et al., 2018; Kim et al., 2020; Sharma et al., 2020b; Hong et al., 2021; Chen et al., 2022; Luo et al., 2023; Moutsoglou et al., 2023; Cao et al., 2024; Chen et al., 2025; Shuai et al., 2025). Dysbiosis and barrier injury may in turn promote the entry of microbial structural components and metabolites into the systemic circulation, amplify systemic inflammation and oxidative stress, and alter the functional states of pulmonary vascular endothelial cells, PASMCs, fibroblasts, and perivascular immune cells. PH-associated intestinal injury and pulmonary vascular pathology may thus form a mutually reinforcing pathological feedback loop.

Tryptophan metabolism may lie at the metabolic intersection of this bidirectional network. Through specific enzyme systems, gut microorganisms can directly convert tryptophan into indole, IPA, IAA, ILA, IAld, indoleacrylic acid, and other metabolites. They may also indirectly regulate host kynurenine and 5-HT metabolism by competing for luminal tryptophan and altering barrier and immune status (Zelante et al., 2013; Cervantes-Barragan et al., 2017; Dodd et al., 2017; Wlodarska et al., 2017; Agus et al., 2018; Roager and Licht, 2018; Sinha et al., 2024). Microbially produced SCFAs can regulate TPH1 expression in enterochromaffin cells and peripheral 5-HT production (Reigstad et al., 2015; Yano et al., 2015), whereas barrier injury and increased inflammatory mediators may induce IDO1 expression in immune or vascular cells and direct more tryptophan into the kynurenine pathway. In other disease models, microbiota-derived IAld can influence host 5-HT metabolism through the AHR–TPH1 pathway (Zelante et al., 2024). This evidence suggests that microbiota-derived indole metabolites may participate in cross-regulation among host tryptophan metabolic pathways, although this mechanism remains to be tested in PH.

Once in the circulation, tryptophan metabolites may exert different effects on the pulmonary vasculature. 5-HT promotes pulmonary vasoconstriction and PASMC proliferation (Morecroft et al., 1999; Eddahibi et al., 2001; Marcos et al., 2004; Morecroft et al., 2005; Eddahibi et al., 2006; MacLean, 2018; Song et al., 2021), whereas the vascular effects of the kynurenine pathway appear to depend on the cellular source, metabolic context, and duration of pathway activation (Jasiewicz et al., 2016; Nagy et al., 2017; Cai et al., 2022; Simpson et al., 2023; Cai et al., 2025). These differences suggest that individual branches of tryptophan metabolism may contribute to pulmonary vascular remodeling in distinct, and sometimes opposing, ways. Microbiota-related indole metabolism most directly reflects the metabolic link within the gut–lung axis, but evidence in PH remains limited. Microbiome and metabolomic studies have identified changes in microbial tryptophan metabolism and indole-related metabolites in patients with PH and animal models (Shuai et al., 2025). AHR studies support the involvement of tryptophan-derived ligands in pulmonary vascular disease but cannot confirm that their effects arise specifically from gut-derived indole metabolites (Dean et al., 2018; Masaki et al., 2021). A recent study in a PH-LHD animal model showed that IPA inhibited PASMC proliferation and improved disease-related phenotypes (Wu et al., 2026). In other disease models, IPA, ILA, and IAld have shown barrier-protective, immunoregulatory, and anti-inflammatory effects (Zelante et al., 2013; Cervantes-Barragan et al., 2017; Wlodarska et al., 2017; Sári et al., 2020; Zhuang et al., 2023; Wang et al., 2024).

A complete causal chain linking a specific microbial taxon, a defined tryptophan-derived metabolite, a pulmonary vascular target cell, and a remodeling phenotype has not yet been established. Future studies combining stable-isotope tracing with gnotobiotic models may help address this gap by distinguishing microbial from host metabolic contributions and testing whether defined microbial strains are necessary and/or sufficient for metabolite production and pulmonary vascular phenotypes.

### 6.2 Therapeutic targeting of the gut microbiota–tryptophan metabolism axis in pulmonary hypertension

Current treatment of PH mainly comprises management of the underlying disease, supportive therapy, and pulmonary vascular-targeted therapy guided by PH classification. Although these measures can improve symptoms, exercise capacity, hemodynamics, and clinical outcomes in some patients, no generally effective means is available to reverse established abnormal vascular cell proliferation, metabolic reprogramming, and structural remodeling (Humbert et al., 2022; Mocumbi et al., 2024). Because the gut microbiota–tryptophan metabolism axis involves intestinal barrier function, immune and inflammatory responses, pulmonary vasomotor regulation, and vascular wall cell proliferation, it may offer new complementary targets for limiting pulmonary vascular remodeling. The principal therapeutic interventions evaluated in PH-related experimental or clinical studies are summarized in **Table 3**, while their potential mechanistic relationships are illustrated in **Figure 4**.

**Table 3:** Therapeutic interventions targeting the gut microbiota–tryptophan metabolism axis in pulmonary hypertension.

Intervention	Study population or model	Main findings	Evidence category	Reference
Butyrate supplementation	Rats with chronic hypoxia-induced PH	Attenuated pulmonary vascular inflammation and remodeling and reduced right ventricular hypertrophy	Preclinical evidence	(Karoor et al., 2021)
Probiotic supplementation (*L. reuteri* DSM 17938)	Juvenile rats with postnatal growth restriction-associated PH	Attenuated PH and right ventricular hypertrophy, supporting a potential role of probiotic modulation of the gut–lung axis	Preclinical evidence	(Wedgwood et al., 2020b)
FMT	Mice with chronic hypoxia-induced PH; patients with PAH	FMT from ACE2-overexpressing mice attenuated PH and associated gut dysbiosis; early clinical studies indicate that microbiota transplantation is feasible and has an acceptable safety profile in PAH, although therapeutic efficacy remains to be established	Preclinical evidence/early clinical feasibility	(Oliveira et al., 2022; Moutsoglou et al., 2025)
Broad-spectrum antibiotics	Rats with SU5416/hypoxia-induced PH	Gut microbiota depletion or restructuring attenuated PH-related phenotypes; however, the intervention is nonselective and may disrupt beneficial microbial communities	Preclinical evidence	(Sanada et al., 2020)
IDO1 inhibition	Rats with MCT-induced PH	Attenuated pulmonary vascular wall thickening, perivascular inflammation, and right ventricular remodeling; the disease-relevant cellular source of IDO1 remains unresolved	Preclinical evidence	(Cai et al., 2025)
TPH1 inhibition	Rats with MCT- or SU5416/hypoxia-induced PH	Pharmacological TPH1 inhibition improved pulmonary hemodynamics and attenuated pulmonary vascular remodeling; inhaled TPH1 inhibition also improved established experimental PAH and right heart dysfunction	Preclinical evidence	(Aiello et al., 2017; Legchenko et al., 2025)
Systemic peripheral TPH1 inhibition (rodatristat ethyl)	Patients with PAH in the randomized phase 2b ELEVATE-2 trial	Reduced peripheral 5-HT synthesis but produced an unfavorable pulmonary hemodynamic response, including increased PVR relative to placebo, indicating that systemic peripheral TPH1 inhibition was not clinically beneficial	Negative clinical trial	(Sitbon et al., 2024)
IPA supplementation	Rats with doxorubicin-induced PH-LHD and PASMCs	Inhibited PASMC proliferation and attenuated pulmonary vascular remodeling through mechanisms associated with Adora1–Akt–mTOR signaling; direct therapeutic evidence is currently limited to PH-LHD	Preclinical evidence	(Wu et al., 2026)

FMT, fecal microbiota transplantation; 5-HT, 5-hydroxytryptamine; IDO1, indoleamine 2,3-dioxygenase 1; IPA, indole-3-propionic acid; MCT, monocrotaline; PAH, pulmonary arterial hypertension; PASMC, pulmonary arterial smooth muscle cell; PH, pulmonary hypertension; PH-LHD, pulmonary hypertension associated with left heart disease; PVR, pulmonary vascular resistance; TPH1, tryptophan hydroxylase 1.

**Figure 4:**
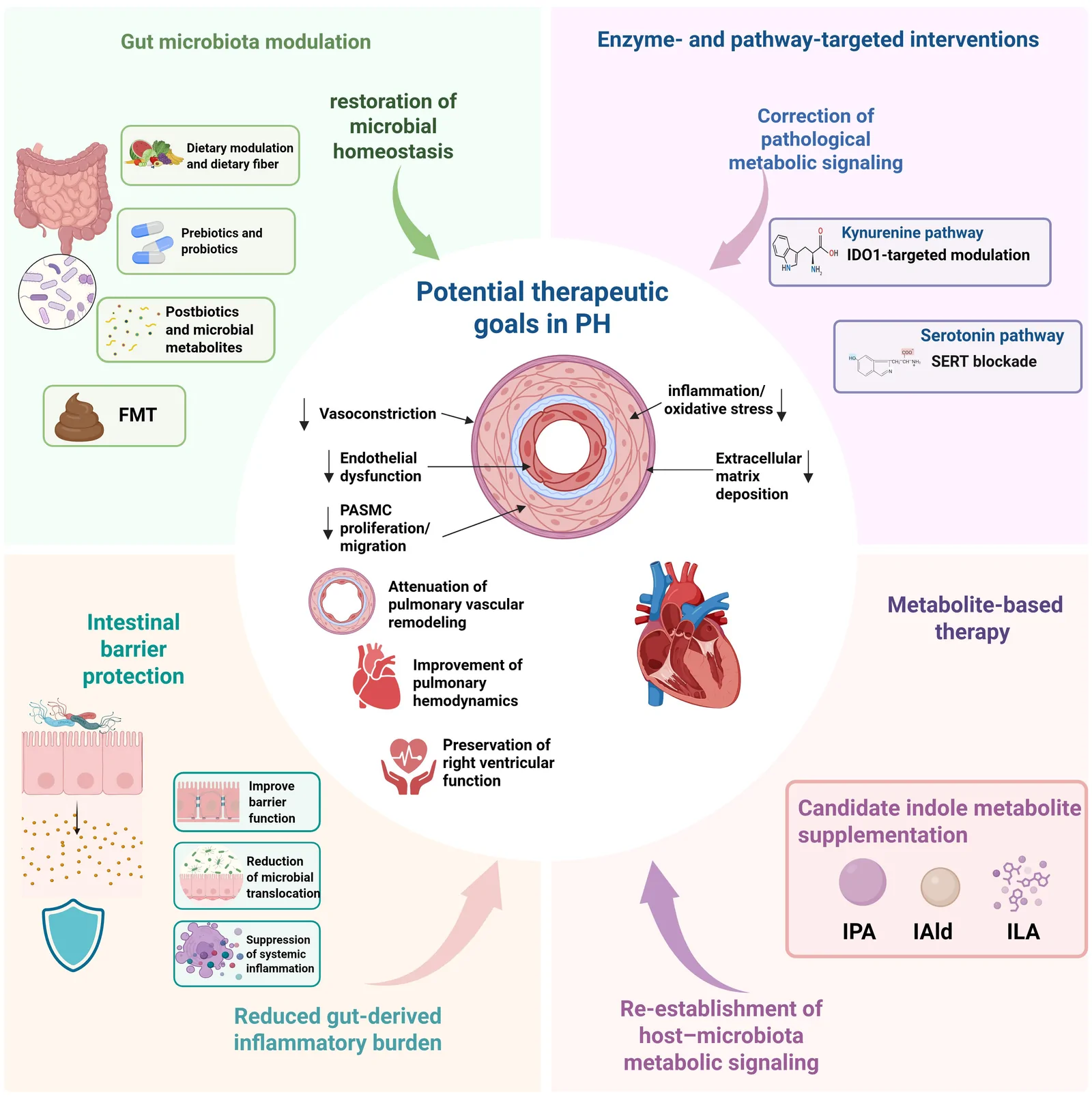
Potential therapeutic strategies targeting the gut microbiota–tryptophan metabolism axis in pulmonary hypertension. Candidate therapeutic strategies can be broadly divided into four interconnected approaches. Gut microbiota modulation through dietary and fiber-based interventions, prebiotics, probiotics, postbiotics, microbial metabolites and fecal microbiota transplantation may help restore microbial homeostasis. Intestinal barrier protection may improve barrier integrity, reduce microbial translocation, and alleviate gut-derived systemic inflammation. Enzyme- and pathway-targeted interventions, including modulation of the IDO1-dependent kynurenine pathway and serotonin signaling, may represent potential therapeutic approaches. Supplementation with candidate indole metabolites, including IPA, IAld, and ILA, may help restore host–microbial metabolic signaling. These strategies may reduce pulmonary vasoconstriction, endothelial dysfunction, PASMC proliferation and migration, inflammation, oxidative stress, and extracellular matrix deposition, thereby attenuating pulmonary vascular remodeling, improving pulmonary hemodynamics, and preserving right ventricular function. Most of these approaches are supported primarily by preclinical evidence, and their efficacy, optimal delivery, and long-term safety in patients with PH remain to be established. FMT, fecal microbiota transplantation; IAld, indole-3-aldehyde; IDO1, indoleamine 2,3-dioxygenase 1; ILA, indole-3-lactic acid; IPA, indole-3-propionic acid; PASMC, pulmonary arterial smooth muscle cell; PH, pulmonary hypertension; SERT, serotonin transporter; ↓, decreased.

Gut microbial composition is influenced by sex, age, diet, lifestyle, disease, medications, and environmental factors. Diet can alter microbial composition and metabolic function and, by changing the intestinal availability of tryptophan, regulate its allocation between host and microbial pathways (Kasperek et al., 2024; Sinha et al., 2024). In PH animal models, butyrate supplementation, selected probiotics, and fecal microbiota transplantation have shown some intestinal barrier-protective and anti-inflammatory effects and have improved pulmonary vascular lesions or right heart phenotypes to varying degrees (Wedgwood et al., 2020b; Karoor et al., 2021; Oliveira et al., 2022; Prisco et al., 2025). Broad-spectrum antibiotics can attenuate PH-related phenotypes in some animal models by depleting or reshaping the gut microbiota (Sanada et al., 2020), but their effects are nonselective and carry risks of disrupting beneficial microbial communities and inducing antimicrobial resistance (Sanada et al., 2020).

Probiotic interventions may also modify PH-related phenotypes by restoring intestinal homeostasis, improving barrier function, and suppressing systemic inflammation. Wedgwood et al. found that *Lactobacillus reuteri* DSM 17938 attenuated PH and right ventricular hypertrophy in juvenile rats with postnatal growth restriction, suggesting that the developing gut–lung axis may influence pulmonary vascular and right heart pathology (Wedgwood et al., 2020b). In rats with MCT-induced PAH, Prisco et al. found that oral *Lactobacillus rhamnosus* reshaped the intestinal bacterial and fungal communities, improved the intestinal barrier, and suppressed systemic inflammation and GP130-related microtubule remodeling in right ventricular cardiomyocytes. This intervention improved right ventricular function but did not significantly reduce the severity of pulmonary vascular disease (Prisco et al., 2025).

Fecal microbiota transplantation (FMT) transfers the fecal microbial community of a screened donor into the recipient gastrointestinal tract to broadly reshape the gut microbial ecosystem. In mice with chronic hypoxic PH, FMT from ACE2-overexpressing mice attenuated PH and associated dysbiosis (Oliveira et al., 2022). Bidirectional transplantation studies further showed that microbiota from patients with idiopathic PAH or from animals with PAH can promote PH-related phenotypes in recipient animals, whereas transplantation of microbiota from healthy donors can attenuate the PH phenotype and restore gut microbial community structure (Shuai et al., 2025). Early clinical studies suggest that microbiota transplantation is feasible and has an acceptable safety profile in patients with PAH, but its efficacy, donor-selection criteria, duration of effect, and long-term risks require evaluation in larger controlled studies (Moutsoglou et al., 2025).

Compared with reshaping the microbial community as a whole, direct modulation of host tryptophan metabolic pathways offers more clearly defined molecular targets. In rats with MCT-induced PH, IDO1 inhibition attenuated pulmonary vascular wall thickening, perivascular inflammation, and right ventricular remodeling (Cai et al., 2025). Chronic hypoxic PH models show that *Tph1* or SERT deficiency attenuates the increase in pulmonary arterial pressure, right ventricular hypertrophy, and pulmonary vascular muscularization, and that targeted knockdown of *Tph1* in pulmonary arterial endothelial cells also improves pulmonary vascular remodeling (Eddahibi et al., 2000; Izikki et al., 2007; Morecroft et al., 2007; Morecroft et al., 2012). In MCT and SU5416/hypoxia rats, pharmacological TPH1 inhibition improves pulmonary hemodynamics and attenuates pulmonary vascular wall thickening, while the inhaled TPH1 inhibitor TPT-004 also attenuates established SuHx-PAH and right heart dysfunction (Aiello et al., 2017; Legchenko et al., 2025). Models involving SERT deficiency, smooth muscle cell-specific SERT overexpression, and 5-HT_2_B receptors on bone marrow-derived cells further support the value of targeting 5-HT synthesis, transport, and receptor signaling (Eddahibi et al., 2000; Guignabert et al., 2006; Launay et al., 2012). In the ELEVATE-2 trial, however, systemic peripheral TPH1 inhibition with rodatristat ethyl reduced indices of 5-HT synthesis but markedly worsened PVR and adversely affected pulmonary hemodynamics and cardiac function (Sitbon et al., 2024). This finding highlights the importance of intervention site and delivery strategy when targeting the serotonin pathway.

Direct supplementation with microbiota-related metabolites is another candidate strategy. A recent study showed that IPA inhibited PASMC proliferation and improved disease-related phenotypes in rats with doxorubicin-induced PH-LHD, and these effects were associated with the Adora1–Akt–mTOR signaling pathway (Wu et al., 2026). This finding provides experimental support for direct supplementation with a microbiota-related indole metabolite, but the evidence remains confined to a single PH-LHD model. The efficacy and safety of IPA in PAH, hypoxia-associated PH, and other PH subtypes require further validation. The barrier-protective, immunoregulatory, and anti-inflammatory effects of IPA, IAld, and ILA in other disease models (Zelante et al., 2013; Cervantes-Barragan et al., 2017; Dodd et al., 2017; Wlodarska et al., 2017; Sári et al., 2020; Zhuang et al., 2023; Wang et al., 2024) provide mechanistic clues to their potential pulmonary vascular effects. Because current evidence in PH is derived mainly from animal studies, future work should define metabolite origin, effective exposure levels, receptor dependence, dose range, and long-term safety.

## 7 Conclusions

A bidirectional relationship may exist between PH and abnormalities of the intestinal microenvironment. PH and right heart dysfunction can promote dysbiosis through intestinal hypoperfusion, venous congestion, and barrier injury; conversely, changes in microbial composition and metabolic function may affect pulmonary vascular homeostasis through microbial products, inflammatory signals, and host–microbiota co-metabolites. Because tryptophan metabolism is regulated by diet, the gut microbiota, host enzymes, and immune status, it may serve as an important metabolic interface linking changes in the intestinal microenvironment to pulmonary vascular remodeling in PH.

The evidence for the three tryptophan metabolic pathways in PH is currently uneven. The 5-HT pathway has relatively extensive support from studies of pulmonary vascular cells and animal models and has also been evaluated clinically. The kynurenine pathway is associated with disease severity and metabolic reprogramming in pulmonary vascular cells, but its effects depend on the specific metabolic branch, cellular source, and disease context. Microbiota-related indole metabolism most clearly reflects the gut–lung axis, but direct evidence in PH remains limited. A complete causal chain linking a specific microbial taxon, a tryptophan metabolite, a pulmonary vascular target cell, and a PH phenotype has not yet been established. It is therefore inappropriate to classify any metabolite or pathway as exclusively protective or pathogenic.

Future studies should combine stable-isotope tracing with gnotobiotic models to define the microbial versus host origins, tissue distribution, and pulmonary vascular target cells of key tryptophan metabolites, and to test whether specific microorganisms are necessary and/or sufficient for metabolite production and pulmonary vascular phenotypes. Longitudinal clinical cohorts and studies across different PH models are also needed to determine whether the relevant microbial and metabolic features are consistently associated with disease progression. On this basis, identifying key strains, metabolites, and enzymatic targets with reproducible effects and developing more tissue- or cell-selective interventions will be necessary to move the gut microbiota–tryptophan metabolism axis from mechanistic research toward patient stratification and precision therapy in PH.
